# 

**Published:** 1921-07-30

**Authors:** 


**July 30, 1921]** **[Price Twopence**

### The Hospital

The Workers' Newspaper of Administrative Medicine and Institutional Life, Administration, National Insurance and Health.

### CONTENTS

**A Newcastle Programme** ... ... ... ... 291

**How Co-operation Must Begin** ... ... ... 292

**Parliament and the Professions** ... ... ... 292

**Notes of the Week** ... ... ... ... ... ... 293

A Lord Lister Memorial—The late Dr. Haultain—A Success for Economy at Cardiff—Irish Hospitals and Medical Secrecy—A Week's Holiday for Five Shillings—The Value of Asylum Visitors—Paying Patients in Poor-Law Hospitals—Voluntary Hospitals in Sussex—Another Hospital Experiment—St. Thomas's and the Financial Stringency—Wider Representation at Sheffield—Swansea's Lead.

**Protein Injection in Treatment** ... ... ... 295

A Feature of Recent Progress.

**The Obligation of Conduct** ... ... ... ... 296

Child Welfare at Brussels.

**Legacies and the Hospital Budget** ... ... ... 297

The Testamentary Habits of Legators.

**The British Hospitals Association** ... ... ... 298

The Record of an Active Year.

### CONTENTS

**The Institutional Library** ... ... ... ... 299

Medical Conduct and Practice—Diseases of the Nervous System—Lippincott's Quick Reference Book for Medicine and Surgery—Barrier Chart for Health Officers—Practical Surgical Nursing for Probationers.

**The Hospital Debates** ... ... ... ... ... 300

**The Public Well-Being** ... ... ... ... ... 301

Some Public-Health Questions Discussed—The Derelict Child and the State.

**The Hospital Week and Pageant** ... ... ... 303

Executive Committee Formed.

**The Editor's Letter-Box** ... ... ... ... ... 304

Diplomas in Radiography—Non-Residential Preliminary Schools.

**The International Union Against Tuberculosis** ... 304

**The Matrons' and Sisters' Department** ... ... 305

The New Territorial Force Nursing Service.

**Round the Hospitals** ... ... ... ... ... 305

**The College of Nursing (Limited by Guarantee)** 308

**Passing Events and Topics** ... ... ... ... 308

